# Rational Design for Enhanced Acyltransferase Activity in Water Catalyzed by the *Pyrobaculum calidifontis* VA1 Esterase

**DOI:** 10.3390/microorganisms9081790

**Published:** 2021-08-23

**Authors:** Amanda Staudt, Henrik Terholsen, Jasmin Kaur, Henrik Müller, Simon P. Godehard, Ivaldo Itabaiana, Ivana C. R. Leal, Uwe T. Bornscheuer

**Affiliations:** 1 Department of Biotechnology & Enzyme Catalysis, Institute of Biochemistry, University of Greifswald, 17487 Greifswald, Germany; amanda_staudt@hotmail.com (A.S.); henrik.terholsen@uni-greifswald.de (H.T.); jasmin.kaur@stud.uni-greifswald.de (J.K.); henrikmuller1301@gmail.com (H.M.); simon_godehard@icloud.com (S.P.G.); 2 Laboratory of Natural Products and Biological Assays, Department of Natural Products and Food, Faculty of Pharmacy, Federal University of Rio de Janeiro, Rio de Janeiro 21941-902, Brazil; ivanafarma@yahoo.com.br; 3 Laboratory of Technological Biochemistry and Biocatalysis, Department of Biochemical Engineering, School of Chemistry, Federal University of Rio de Janeiro, Rio de Janeiro 21941-909, Brazil; ivaldoufrj@gmail.com

**Keywords:** PestE, acyltransferase, protein engineering, biocatalysis, acyl transfer, transesterification, monoterpene acylation

## Abstract

Biocatalytic transesterification is commonly carried out employing lipases in anhydrous organic solvents since hydrolases usually prefer hydrolysis over acyl transfer in bulk water. However, some promiscuous acyltransferases can catalyze acylation in an aqueous solution. In this study, a rational design was performed to enhance the acyltransferase selectivity and substrate scope of the *Pyrobaculum calidifontis* VA1 esterase (PestE). PestE wild type and variants were applied for the acylation of monoterpene alcohols. The mutant PestE_I208A is selective for (–)-menthyl acetate (E-Value = 55). Highly active acyltransferases were designed, allowing for complete conversion of (–)-citronellol to citronellyl acetate. Additionally, carvacrol was acetylated but with lower conversions. To the best of our knowledge, this is the first example of the biocatalytic acylation of a phenolic alcohol in bulk water. In addition, a high citronellol conversion of 92% was achieved with the more environmentally friendly and inexpensive acyl donor ethyl acetate using PestE_N288F as a catalyst. PestE_N288F exhibits good acyl transfer activity in an aqueous medium and low hydrolysis activity at the same time. Thus, our study demonstrates an alternative synthetic strategy for acylation of compounds without organic solvents.

## 1. Introduction

Hydrolytic enzymes are versatile biocatalysts with many industrial applications, due to their broad substrate scope, absence of cofactor requirements, stability in organic solvents, and good chemo-, regio-, and stereoselectivity [1,2,3]. Usually, these enzymes are applied for the hydrolysis of esters, amides, or lipids in aqueous systems, since water acts as a nucleophile in the reaction [4,5]. Still, these enzymes can also act as biocatalysts for condensation reactions and in alcoholysis [1].

The capacity of some hydrolases, especially lipases, to synthesize esters and amides under anhydrous conditions increases the applicability of these enzymes [1,6,7]. Nevertheless, hydrolases typically favor a hydrolytic reaction over acyl transfer in water [8,9,10,11], limiting the direct acylation of interesting substances found naturally in aqueous raw materials. 

In contrast to conventional hydrolases, some promiscuous hydrolases/acyltransferases are able to catalyze acyl transfer in aqueous systems, presenting an opportunity for cascade reactions in aqueous solutions. This is also more environmentally friendly than ionic liquids [12] or often hazardous and expensive organic solvents [5,13], which are then no longer required. Examples of promiscuous acyltransferases are enzymes from the CAL-A superfamily [14], an aryl esterase from *Mycobacterium smegmatis* (MsAcT) [13,15,16], family VIII carboxylesterases [17], and the enzymes from the bacterial hormone-sensitive lipase (bHSL) family [5,18].

Due to their acyltransferase activity in water, a lipase from Sphingomonas sp. (SpL) and the lipase A from Pseudozyma antarctica (CAL-A) were used for amide and ester synthesis in the presence of 4% and 50% water, respectively [19,20]. Regarding cascade reactions, the acyltransferase MsAcT was applied in a combination with a transaminase to synthesize *N*-benzylacetamide from benzaldehyde [21]. Furthermore, MsAcT was investigated in our group, and we observed approximately 50% conversion in the synthesis of benzyl acetate in a transesterification reaction between vinyl acetate and benzyl alcohol at an equimolar ratio in an aqueous environment [13].

Recently, our group also discovered esterase Est8 as a promiscuous hydrolase/acyltransferase. Est8 was the first enzyme from the bacterial hormone-sensitive lipase (bHSL) family, for which promiscuous acyltransferase activity was described [18]. Subsequently, we developed a sequence-based prediction method for acyltransferase activity, demonstrating that the active site hydrophobicity is directly related to the acyltransferase activity towards non-polar acyl acceptors. This analysis made it possible to identify and biochemically characterize five bHSLs with high acyltransferase activity [5].

One of the most promising acyltransferases identified in the study by Müller et al. [5] was the *Pyrobaculum calidifontis* VA1 esterase (PestE). This carboxylesterase (PDB code: 3ZWQ), first reported by Hotta et al. [22], is a highly thermostable biocatalyst. It has been shown to catalyze the hydrolysis of bulky substrates [22,23] and notably presents activity towards tertiary alcohols in transesterification reactions in organic solvents [6], which was associated with the high hydrophobicity inside the substrate-binding pocket [5,24]. Recognizing the high potential of this enzyme, in this work, we performed a rational design to enhance its acyltransferase activity for the biocatalytic synthesis of monoterpene esters in an aqueous solution without organic solvents.

## 2. Materials and Methods

### 2.1. Material

(±)-Linalool (97%), (±)-menthol (≥ 98.0%), (±)-citronellol (analytical standard), carvacrol (99%), (–)-menthol (99%), and vinyl acetate were purchased from Sigma-Aldrich. All other chemicals and solvents were purchased from Sigma, VWR, or Carl Roth. The synthetic gene encoding PestE for expression in *Escherichia coli*—subcloned into the pET-21a vector—was based on the gene reported by Hotta et al. [22].

### 2.2. Gene Expression and Protein Purification

Chemically competent *E. coli* BL21(DE3) cells were transformed with expression vectors by heat shock followed by cooling and then plated on LB agar containing 50 µg·mL^−1^ ampicillin. Pre-cultures (4 mL LB containing 50 µg·mL^−1^ ampicillin) were inoculated with single colonies and incubated overnight (37 °C, 180 rpm). LB medium (200 mL containing 50 µg·mL^−1^ ampicillin) was inoculated with 0.1% (*v/v*) of the pre-culture and incubated (37 °C, 180 rpm) until it reached an OD_600_ of 0.6. Protein expression was induced by the addition of isopropyl-β-D-thiogalactopyranoside (IPTG) to a final concentration of 0.4 mM followed by incubation for ~20 h at 20 °C at 180 rpm.

Cells were harvested by centrifugation at 4000× *g* and 4 °C for 15 min, and the cell pellets were resuspended with 4 mL equilibration buffer (50 mM potassium phosphate, 300 mM sodium chloride, 10 mM imidazole, pH 8.0). Cells were disrupted by sonication on ice (two cycles of 5 min sonication (30% intensity, 50% pulsed cycle)) using a SONOPULS HD 2070 (BANDELIN Electronic GmbH & Co. KG, Berlin, Germany), and the lysates were clarified by centrifugation at 10,000× *g* and 4 °C for 30 min. For purification, the crude lysates were applied to 1.5 mL Roti^®^ Garose-His/Ni Beads (Carl Roth, Karlsruhe, Germany). The resins were washed with 15 mL washing buffer (50 mM sodium phosphate, 300 mM sodium chloride, 20 mM imidazole, pH 8.0) before target proteins were eluted with elution buffer (50 mM sodium phosphate, 300 mM sodium chloride, 250 mM imidazole, pH 8.0). Elution fractions were treated at 80 °C for 20 min (500 rpm), centrifuged (17,000× *g*, 4 °C for 5 min), and the supernatant transferred to the storage buffer (50 mM potassium phosphate, 300 mM sodium chloride, pH 8.0) using PD-10 desalting columns (GE Healthcare, Chalfont St Giles, UK). Protein concentrations were determined at 280 nm using a NanoDrop™ 1000 spectrophotometer (ThermoFisher, Darmstadt, Germany), while the purity of the proteins was investigated by SDS-PAGE. The purified enzymes were mixed with 0.1% (*v/v*) Triton-X-100 for storage at 4 °C.

### 2.3. In Silico Methods

Structural analysis and molecular modeling experiments of the structure of PestE (PDB entry: 3ZWQ) were performed using YASARA (Vienna, Austria) [25] and UCSF Chimera (San Francisco, CA, USA) [26]. The substrate-binding sites in the PestE crystal structure were identified and analyzed using the VINA docking tool [27] of the YASARA software. Molecular modeling was performed by two different approaches: the first was by evaluating the residues responsible for the water network and changing the structure to minimize hydrolase activity. The second was by increasing the hydrophobicity using the Chimera software with visualization of hydrophobic and hydrophilic regions.

### 2.4. Site-Directed Mutagenesis

Variants were constructed using the Q5^®^ Site-Directed Mutagenesis Kit (New England Biolabs GmbH, Ipswich, UK). Non-overlapping DNA-oligonucleotides were designed using the online NEBaseChanger tool for the mutations: H95A: forward primer (5′→3′): CGTGGAGACTgcgGACCACGTGTGTAGGC; reverse primer (5′→3′): CTCCCCAA GACGAAGCCC; I208A: forward primer (5′→3′): CGAATACGTCgcg CTCACCGCCGACTTAATGG; reverse primer (5′→3′): GGCCCGCTGTACTCCACT; N288F: forward primer (5′→3′): CGGCTTCGTCtttTTCTACCCCATATTAGAAG, reverse primer (5′→3′): TGGATGACGCCGTTGTAC. PCR amplification and KLD reactions were performed according to the manufacturer’s protocol. The correct introduction of the desired mutations was confirmed by sequencing by Eurofins Genomics GmbH (Ebersberg, Germany).

### 2.5. SDS-PAGE Analysis

The protein purity was analyzed by SDS-PAGE. Purified proteins were denatured by heating (95 °C, 10 min) in a 10% (*w/v*) sodium dodecyl sulfate (SDS) solution followed by centrifugation at 13,000× *g* for 5 min. The proteins were separated on 12.5% acrylamide gels at a constant voltage of 200 V. A protein standard was added for protein size comparison (Pierce^TM^ Unstained Protein MW Marker, ThermoFisher, Darmstadt, Germany) and stained by Coomassie Brilliant Blue G-250.

### 2.6. Biocatalytic Experiments

Purified PestEs (wild type and variants) were applied for transesterification of linalool, menthol, carvacrol, and citronellol. For the reactions, a mixture of 20 mM monoterpene (from a 1 M stock in acetonitrile), vinyl acetate at a molar ratio of 1:10 (monoterpene:acyl donor), 0.1% (*v/v*) Triton-X-100 (from a 10% (*v/v*) stock in water), and 0.2 µg·mL^−1^ of the purified PestE variants were adjusted to a total volume of 1 mL with buffer (50 mM potassium phosphate, 300 mM sodium chloride, pH 8.0). Reactions were performed in 1.5 mL reaction tubes and incubated at 40 °C and 1000 rpm in a ThermoMixer Comfort (Eppendorf AG, Hamburg, Germany). Reactions without enzymes were performed to determine background transesterification and served as control. Time samples (10 µL) were taken, quenched with 10 µL of 2 M HCl, and extracted with 200 µL ethyl acetate (EtOAc). After drying with anhydrous MgSO_4_, samples were analyzed by GC. In order to evaluate the possibility of producing monoterpene esters with a more environmentally friendly and cheaper acyl donor, reactions were also performed for (±)-citronellol with ethyl acetate as acyl donor. The reaction conditions were described above, with 20 µg·mL^−1^ of the purified PestE variants.

### 2.7. GC Analysis

Reactions with linalool, carvacrol, and citronellol were analyzed by GC-MS using a GC-QP2010 SE (Shimadzu, Kyoto, Japan) equipped with a ZB-5MSi column (30.0 m × 0.25 mm, 0.25 μm film thickness, Phenomenex, Torrance, CA, USA). Injector temperature was 220 °C, a flow rate of 1.20 mL·min^−1^ was used, and 1 µL sample at a split ratio of 10 was injected. The column temperature was held at 80 °C for 3 min, increased to 260 °C at 10 °C·min^−1^, totalizing a 21 min method. Mass spectrum ion source and interface temperature was 220 °C, and the identification started after 3 min of the run.

Biocatalysis reactions with menthol were analyzed by GC-MS using a GC-QP2010 SE (Shimadzu, Kyoto, Japan) equipped with a β-TBDAc column (25.0 m × 0.25 mm, Macherey-Nagel, Düren, Germany). Injector temperature was 220 °C, a flow rate of 2.06 mL·min^−1^ was used, and 1 μL sample at a split ratio of 30 was injected. The column temperature was held at 80 °C for 5 min, increased to 145 °C at 1.75 °C·min^−1^, held for 5 min, and then increased to 180 °C at 20 °C·min^−1^ and kept for 6.11 min, resulting in a 55 min method. Mass spectrum ion source and interface temperature were 220 °C. 

## 3. Results

An initial study regarding menthol acylation with known hydrolases/acyltransferases was performed in order to verify potential acyltransferases for monoterpene ester synthesis. For this, the enzymes PestE, 1EVQ (Est2 from *Alicyclobacillus acidocaldarius*), CAL-A (lipase A from *Pseudozyma antarctica*), and PLE-6 (Pig Liver Esterase 6) were applied as catalysts. PestE showed the highest activity in menthol acylation (Figure S1), achieving 81% of the esterified product but with undesired subsequent hydrolysis. This behavior is known as an obstacle for biocatalytic ester production in water, in view that the optimum time point for the highest conversion needs to be determined to stop the reaction immediately; this makes enzymatic acyl transfer reactions in water somewhat challenging [5]. In addition, the possibility of enabling enantioselective acylation of (±)-menthol by mutagenesis has been investigated.

**Figure 1:**
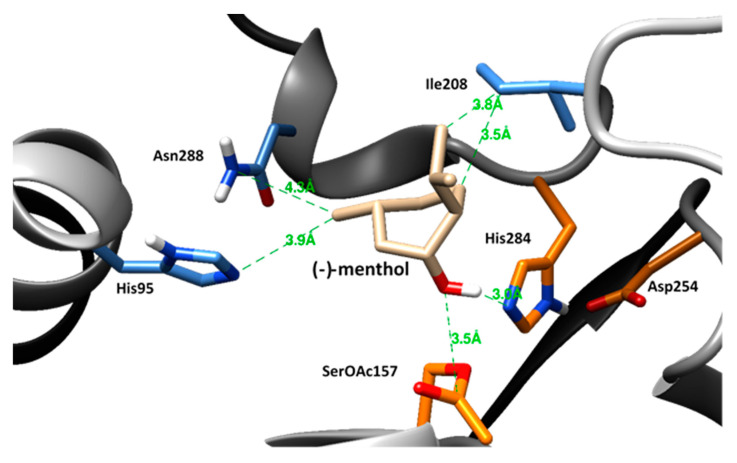
Interaction network for (–)-menthol within PestE (PDB code: 3ZWQ, [24]) based on a substrate docking. Residues interacting with (–)-menthol are shown in light blue, residues of the catalytic triad Ser157, His284, Asp254 are in orange. The model was created based on an acetylated Ser157.

Aiming to decrease product hydrolysis, PestE was used as an object for the rational design. To identify target residues for site-directed mutagenesis, molecular docking was performed using (–)-menthol as a model substrate to study its binding in the PestE active site, as shown in Figure 1.

Residues composing the substrate binding of (–)-menthol in the active site of PestE were examined. Three residues were selected, His95, Ile208, and Asn288, to perform rational protein engineering aiming to increase active site hydrophobicity and/or tunnel size. To enhance the hydrophobicity, the residues His95 and Asn288 were selected, while Ile208 was selected to increase the tunnel size. The increase in the active site region hydrophobicity can promote a more favorable surrounding for organic nucleophiles than for water. Moreover, the tunnel is the cavity space that connects the protein surface to the active site, and the residues forming this tunnel can have a significant influence on the biocatalytic properties so that the decrease in the residue size in these positions can increase the acceptance of larger substrates and the flux of substrates and products [28].

**Figure 2:**
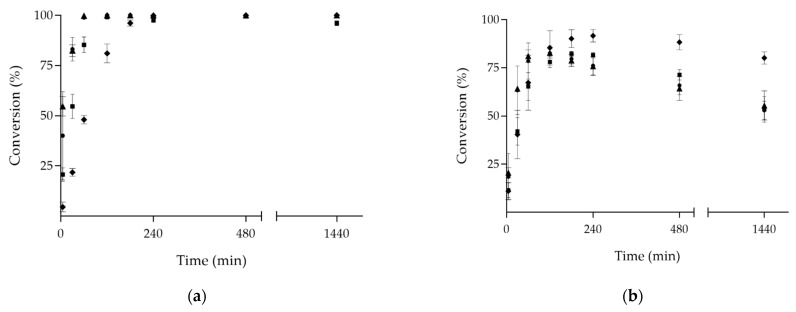
Enzymatic acylation of (±)-citronellol over time with (**a**) vinyl acetate and (**b**) ethyl acetate as acyl donors. Where: (●) PestE_wt; (■) PestE_H95A; (▲) PestE_I208A; (▼) PestE_N288F. Reaction conditions: Molar ratio 1:10 ((±)-citronellol:acyl donor), using 20 mM (±)-citronellol, 0.1% (*v/v*) Triton-X-100, 20 μg·mL^−1^ of the purified enzymes when ethyl acetate was used, or 0.2 μg·mL^−1^ of the purified enzymes when vinyl acetate was used, in 1 mL of an aqueous buffer (50 mM potassium phosphate, 300 mM sodium chloride, pH 8.0) at 1000 rpm and 40 °C. Reactions were conducted in triplicate, and the average values are shown. Reactions without enzymes did not result in ester products.

PestE wild type and variants were purified by a two-step approach consisting of affinity chromatography followed by heat precipitation. The purity of the purified enzymes was evaluated by SDS-PAGE (Figure S2). Subsequently, several monoterpenes were used as model compounds to determine the acyltransferase activity of PestE enzymes towards these primary (citronellol—Figure 2a and Table S1), secondary (menthol—Table 1), tertiary (linalool—data not shown), and phenolic (carvacrol—Table S3) alcohols with vinyl acetate as an acyl donor. In all reactions, 0.1% (*v/v*) Triton-X-100 was added because previous studies found that Triton-X-100 prevented the protein instability that occurs at low concentrations of purified PestE [22].

The results revealed that only the tertiary alcohol linalool was not converted, despite the efforts to increase the substrate entrance space, possibly due to the high steric hindrance and less reactivity of this tertiary alcohol. Reactions with primary and secondary alcohols showed similar conversions for all variants, although the conversion rate was lower for PestE_N288F. It is also obvious that the variant PestE_N288F decreased the undesired product hydrolysis (Table S1 and Table 1). Regarding the phenolic alcohol carvacrol, low conversions were obtained for all PestE variants, still, this is the first time a promiscuous acyltransferase was reported to acylate a phenolic alcohol in an aqueous environment (Table S3).

Table 1 shows that all investigated variants catalyze the conversion of menthol to menthyl acetate with similar maximum conversions. The results (Table 1) confirmed low enantioselectivity for the wild type and PestE_H95A, and moderate E-values for the PestE_N288F mutant. Contrary to this, PestE_I208A is highly enantioselective for the acetylation of (–)-menthol, showing an enantiomeric excess of 94%*ee* and an E-value over 55. Additionally, the initial acylation is higher for PestE_I208A compared to the wild type. These results suggest that the increase of the tunnel size by rational design facilitated the access of (–)-menthol to the enzyme’s active site.

Transesterification reactions with ethyl acetate, a much less activated acyl donor compared to vinyl acetate, are reversible and, hence, could lead to lower conversion to esterified products. As all the PestE variants were shown to be efficient biocatalysts for the acylation of citronellol, this substrate was used in reactions with ethyl acetate. The results, shown in Figure 2b and Table S2, demonstrate that all PestE variants studied can perform the acylation with ethyl acetate as an acyl donor almost equally as well as with vinyl acetate. The PestE_N288F variant enabled higher conversion (92% ester formed within 4 h) in comparison to the other acyltransferase variants, where a maximum of 83% was observed together with subsequent hydrolysis of the ester product (Table S2). This finding could be linked to a potential reorganization of the water network (Figures S3–S5). Thus, the undesired and commonly observed hydrolysis of ester products was slowed down, which can be explained by its disrupted water network (Figure S5), decreasing the hydrolytic activity.

## 4. Discussion

In this study, we performed rational protein engineering of the *Pyrobaculum calidifontis* VA1 esterase (PestE) using two different concepts, the first to increase the hydrophobicity of the active site, with the mutations H95A and N288F, aiming to disrupt the water network and to increase affinity to organic nucleophiles. The second approach was to increase the tunnel size by the mutation I208A. Interestingly, the increased hydrophobicity with the mutations H95A and N288F did not increase the acyltransferase activity, although it is believed that increased hydrophobicity facilitates binding of (non-polar) acyl acceptors [5,15,30]. However, the undesired subsequent hydrolysis of the acylated product was substantially reduced by the variant PestE_N288F, which showed very high up to complete conversions to the ester products and reduced subsequent hydrolysis. The reduced hydrolysis could be due to inactivation of the water molecules in the active site by the introduction of phenylalanine (Figure S5). In the same way, the variant PestE_H95A showed higher hydrolysis as the water network was strengthened (Figure S4), although the hydrophobicity increased. Thus, the reorganized water network in the active site seems to affect hydrolysis activity stronger than the increase in hydrophobicity. For completeness, it has to be mentioned that the PestE_N288F variant presented higher electrophoretic mobility (Figure S2), which might be caused by protein digestion; nevertheless, this variant still showed very high activity. Meanwhile, the variant I208A is highly selective on the (–)-menthol acylation, which can be related to a better access or a better binding of the substrate into the enzyme active site. Again, the decreased hydrophobicity did not decrease but increased the acyltransferase activity in the mutant PestE_I208A, emphasizing that factors such as the access to the binding site are, in this particular case, more relevant for the conversion of monoterpene substrates. Consequently, the highest conversion of the challenging substrate carvacrol was achieved with PestE_I208A, demonstrating not only acyltransferase activity on phenolic alcohols for the first time, but also that enzyme activity is limited by access to the active site. In the same manner, the conversion of tertiary alcohols is eventually only limited by the accessibility of the active site. These findings might help to develop effective acyltransferases for further applications. The outstanding enzymatic activities of the PestE variants for good substrates as citronellol underline the synthetic potential of acyltransferase-catalyzed acylation reactions in aqueous solutions. Even though the protocol presented in this paper requires the addition of Triton-X-100 for the protein stabilization [22], in experiments with heat shock-enriched lysate (without affinity chromatography purification, data not shown) this was not necessary, which can facilitate the enzyme application. Furthermore, we could show that the acyl donor vinyl acetate can be replaced by the less toxic and more environmentally friendly ethyl acetate, in relation to vinyl acetate [31], still leading to high conversions at relatively short reaction times.

## 5. Conclusions

In this study, novel PestE variants have been designed for the acylation of monoterpene in water. All variants represented high activity for citronellol. PestE_I208A presented high enantioselectivity for the acetylation of (–)-menthol. PestE_N288F showed good acyl transfer in water, reducing the commonly observed hydrolysis of the monoterpene esters formed upon prolonged reaction times. This variant also catalyzes acylation successfully with the cheaper and environmentally friendly acyl donor ethyl acetate. With carvacrol, a phenolic alcohol was acylated with a promiscuous acyltransferase for the first time.

## Figures and Tables

**Table 1:** Menthyl acetate conversion and product enantiomeric excess from transesterification reactions catalyzed by PestE wild type and mutants.

Time (min)	PestE_wt		PestE_H95A		PestE_I208A		PestE_N288F	
	Conv (%)	*%ee* (E)	Conv (%)	*%ee* (E)	Conv (%)	*%ee* (E)	Conv (%)	*%ee* (E)
10	0	-	0	-	12	100	0	-
30	7	74	3	67	17	100	3	100
60	18	67	8	81	31	95 (59)	9	97
120	32	59 (5)	24	66	36	94 (55)	20	89
180	37	58 (5)	31	62 (6)	38	94 (58)	29	81
240	38	57 (5)	28	66	36	95 (67)	35	79 (13)
480	32	68 (7)	31	71 (8)	24	100	44	80 (17)
1440	19	80	9	67	13	100	38	79 (14)

Conversion (%) and optical purity (%*ee*) were determined from GC-MS analytical data using a chiral column; The E-value was determined according to Rakels, Straathof, and Heijnen [29]. Reaction conditions: molar ratio 1:10 ((±)-menthol:vinyl acetate), using 20 mM (±)-menthol, 0.1% (*v/v*) Triton-X-100, 0.2 μg·mL−1 of the purified enzymes in 1 mL of an aqueous buffer (50 mM potassium phosphate, 300 mM sodium chloride, pH 8.0) at 1000 rpm and 40 °C. Reactions were conducted in triplicate, and the average values are shown. Reactions without enzymes did not result in ester products.

## Data Availability

Data are contained within the article and the supplementary material.

## Supplementary Materials

The following are available online at https://www.mdpi.com/article/10.3390/microorganisms9081790/s1, Figure S1: Enzymatic acylation of (±)-menthol over time with vinyl acetate as acyl donor, Figure S2: SDS-PAGE analysis of samples of the purified PestE (wt and variants) used in this study, Table S1: Conversion (%) of (±)-citronellol and vinyl acetate to citronellyl acetate over time using PestE wild type and mutants as biocatalysts, Table S2: Conversion (%) of (±)-citronellol and ethyl acetate to citronellyl acetate over time using PestE wild type and mutants as biocatalysts, Table S3: Conversion (%) of carvacrol to carvacryl acetate over time using PestE wild type and mutants as biocatalysts, Figure S3: Water network of PestE wild type active site, Figure S4: Water network of PestE_H95A active site, Figure S5: Water network of PestE_N288F active site.
