# Supplementary material for: Rational Design for Enhanced Acyltransferase Activity in Water Catalyzed by the Pyrobaculum calidifontis VA1 Esterase
Source: Microorganisms. 2021 Aug 23;9(8):1790. doi: 10.3390/microorganisms9081790 (PMC8402108; doi:10.3390/microorganisms9081790)
Supplement: Supplementary file 1 [file microorganisms-09-01790-s001.zip › microorganisms-1334706-supplementary.pdf]

Supplementary Material

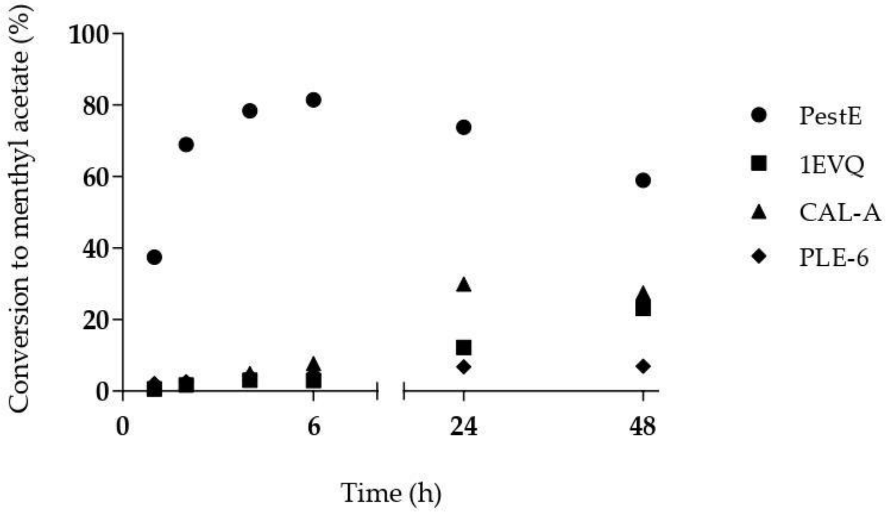

Figure S1. Enzymatic acylation of (±)-menthol over time with vinyl acetate as acyl donor. Reaction conditions: molar ratio 1:100 (alcohol:acyl donor), concentration of 20 mM (±)-menthol in 1 mg lyophilized enzyme lysate per mL aqueous buffer (200 mM potassium phosphate, pH 8.0), 1000 rpm and 40°C, for reactions conducted with PestE, 1EVQ or CAL-A. For reactions conducted with PLE-6 the molar ratio was reduced to 1:10 (alcohol:acyl donor). In control reactions without enzymes no ester product formation was observed.

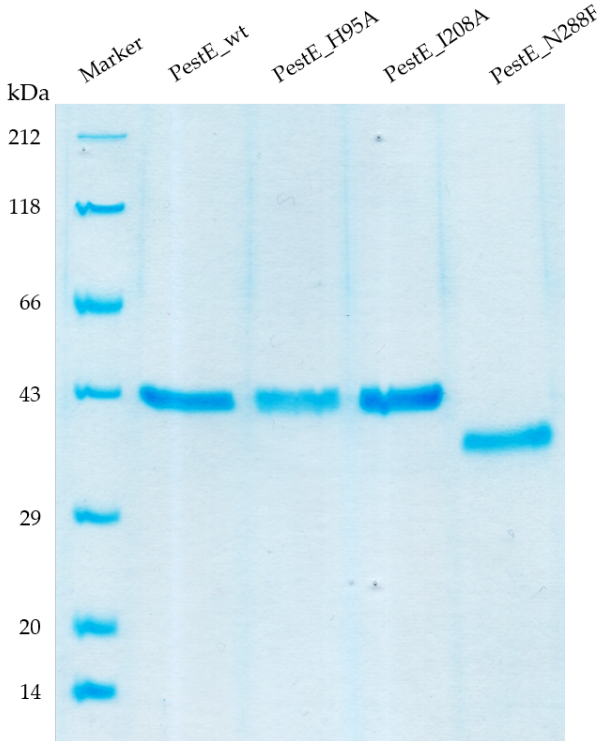

Figure S2. SDS-PAGE analysis of samples of the purified PestE (wt and variants) used in this study. Pierce™ Unstained Protein MW Marker (ThermoFisher, Germany) was used as a reference.

Table S1. Conversion (%) of (±)-citronellol and vinyl acetate to citronellyl acetate over time using PestE wild type and mutants as biocatalysts

Time (min)	PestE_wt	PestE_H95A	PestE_I208A	PestE_N288F
5	40	21	55	5
30	83	55	82	22
60	99	85	99	48
120	100	99	100	81
180	100	100	100	96
240	100	97	100	100
480	100	99	100	100
1440	100	96	100	100

Conversion (%) was determined by GC-MS. Reaction conditions: molar ratio 1:10 ((±)-citronellol:vinyl acetate), using 20 mM (±)-citronellol, 0.1 % (v/v) Triton-X-100, 0.2 $\mu g.mL^{-1}$ of the purified enzymes in 1 mL aqueous buffer (50 mM potassium phosphate, 300 mM sodium chloride, pH 8.0) at 1000 rpm and 40°C. Reactions without enzymes did not show the formation of ester product.

Table S2. Conversion (%) of citronellol and ethyl acetate to citronellyl acetate over time using PestE wild type and mutants as biocatalysts

Time (min)	PestE_wt	PestE_H95A	PestE_I208A	PestE_N288F
5	19	11	21	11
30	63	42	64	40
60	79	65	81	67
120	83	78	83	85
180	80	82	79	90
240	76	82	76	92
480	66	71	64	88
1440	53	53	55	80

Conversion (%) was determined by GC-MS. Reaction conditions: molar ratio 1:10 ((±)-citronellol:ethyl acetate), using 20 mM (±)-citronellol, 0.1 % (v/v) Triton-X-100, 0.2 $\mu g.mL^{-1}$ of the purified enzymes in 1 mL aqueous buffer (50 mM potassium phosphate, 300 mM sodium chloride, pH 8.0) at 1000 rpm and 40°C. Reactions without enzymes did not show the formation of ester product.

Table S3. Conversion (%) of carvacrol to carvacryl acetate over time using PestE wild type and mutants as biocatalysts

Time (min)	Without enz	PestE_wt	PestE_H95A	PestE_I208A	PestE_N288F
5	0	1	<1	1	1
20	0	2	2	2	2
30	0	4	2	5	1
45	< 1	7	6	11	5
60	1	4	4	5	4
120	2	4	3	3	2
240	3	2	2	3	1
1440	2	2	1	1	1

Conversion (%) was determined by GC-MS. Reaction conditions: molar ratio 1:10 (carvacrol:vinyl acetate), using 20 mM carvacrol, 0.1 % (v/v) Triton-X-100, 0.2 $\mu g.mL^{-1}$ of the purified enzymes in 1 mL aqueous buffer (50 mM potassium phosphate, 300 mM sodium chloride, pH 8.0) at 1000 rpm and 40°C.

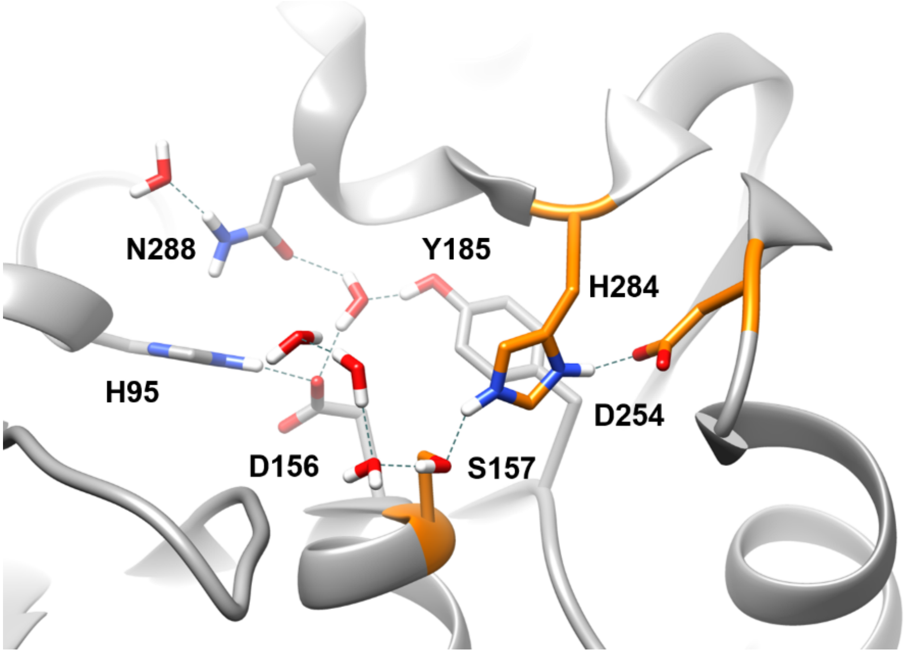

Figure S3. Water network within the PestE wild type active site.

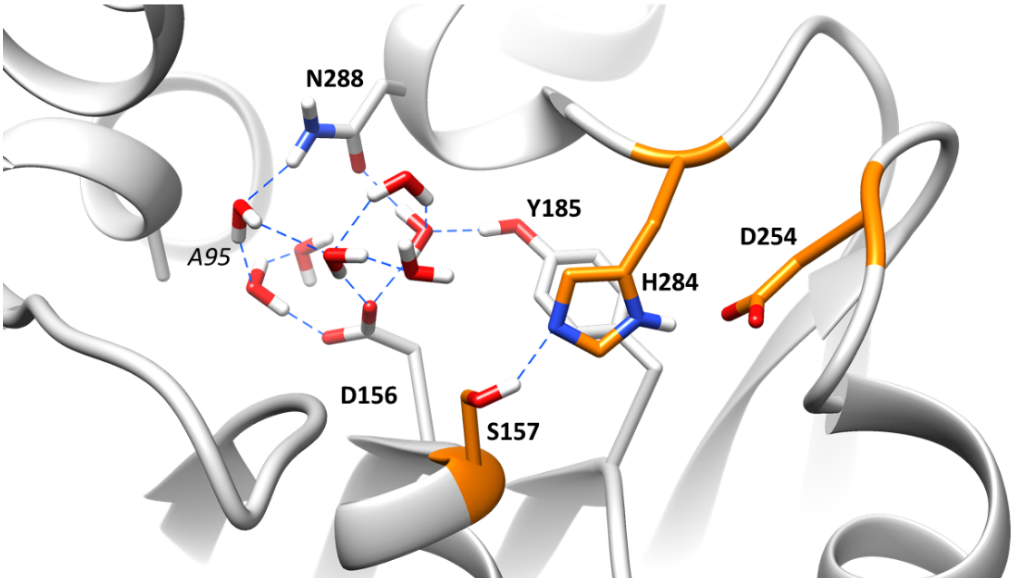

Figure S4. Water network within the PestE_H95A active site. Simulated by Molecular Dynamics with YASARA Software.

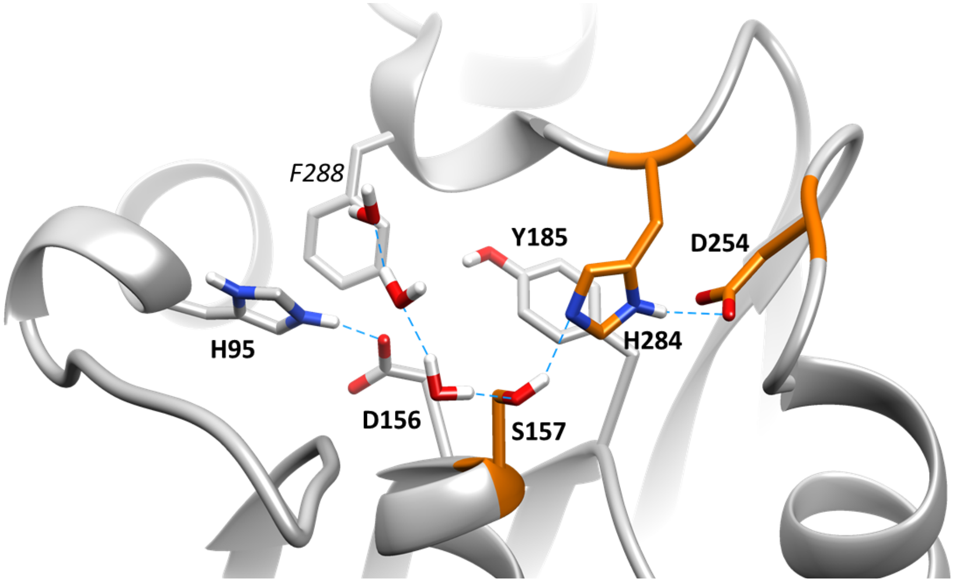

Figure S5. Water network within the PestE_N288F active site. Simulated by Molecular Dynamics with YASARA Software.
